# Indents

**Published:** 1915-07

**Authors:** 


### INDENTS

☞ **Brief Articles of Technical or Scientific Value will receive notice and comment. Contributions invited.**

*Buckley's Desensitizing Paste.* I have just read the interesting but rather discouraging report printed at page 544 of the May *Dental Cosmos,* by Dr. Alfred R. Starr of New York City. The brief report closes with a statement that the writer fears I have made a mistake in claiming so much for the desensitizing paste. If the Doctor's experience with this preparation was that of the hundreds of others who are using the preparation today, I certainly would be forced to admit that I had made a mistake in recommending this preparation as I did in the article which appeared in the December *Items of Interest.* But, so far as I am able to learn, such results are not obtained by the men who are using the remedy as recommended. Judging from Dr. Starr's report, one would be led to believe that he had used the remedy generously in practically all cases in his practice. I am sure that the average practitioner does not use analgesia or conductive anesthesia in the larger percentage of cases; neither did I intend to have desensitizing paste used in every case. The use of all such means, in my opinion, should be confined to those cases of hypersensitive dentin in which the pulp of the tooth is uninvolved, and in which permanent work can not be accomplished without inflicting more pain than we are justified in doing today, with the different methods at our command.

It has also come to my knowledge that a statement was made at the recent meeting of the Michigan State Dental Association to the effect that desensitizing paste will cause death of the pulp in practically every case in which it has been used. In this connection I desire to say that before this preparation was offered to the dental profession it was given every test, including the reliable test of *time,* to ascertain whether or not it was a safe and reliable remedy to be used for purposes for which it was intended. A great deal of experimenting was done by myself in the laboratory, and after the formula was worked out it was given every clinical test both by myself and others. It was found that the remedy will not affect sound dentin to any appreciable depth; and if used in cases where there is sound and healthy dentin intervening between the cavity of decay and the pulp proper, *it is absolutely impossible to devitalize the pulp or affect it deleteriously with the remedy.*

It is an easy matter for those who are still suspicious of the permanent effects of desensitizing paste upon the pulp to determine for themselves whether or not sensation returns in dentin which has once been desensitized by the remedy. If the preparation is applied to and sealed in contact with exposed sensitive areas at the necks of teeth for a period of twenty-four hours or longer, it will be found that the sensitiveness is completely destroyed. It will also be found that in about three weeks' time the sensation will begin to recur, and in five or six weeks the areas in most cases will be as sensitive as they were before the preparation had been used. In fact, the effect of desensitizing paste on sound dentin is so transitory that I have practically abandoned its use for sensitive necks of teeth, preferring to use other means which will produce more permanent results. While the remedy will go through decayed dentin like wildfire and completely destroy all sensitiveness therein, it has been the experience not only of myself, but others, that the remedy will not affect sound dentin to any dangerous depth.

It was never intended that desensitizing paste should be employed in cases of extensive decay, unless used with the idea of subsequently removing the pulp should it be found that the decay had extended to or nearly to the organ. The remedy is recommended for *hypersensitive dentin* where the pulp of the tooth is uninvolved. In such cases it is a specific and its use is absolutely safe.

It seems to me that the test of time is the best one, after all, to give any remedy. It is now about fourteen months since I first requested certain men of the profession to use desensitizing paste in their practice. A word from some of these men will be of interest. I am privileged to herewith submit a voluntary statement from three men whose reliability and sincerity of purpose can not be questioned.

Dr. Hart J. Goslee says: "I have just read in the May *Dental Cosmos* Dr. Starr's history of his experience with desensitizing paste, and have also heard of similar statements being made recently at the Michigan State Dental Association. These experiences are so diametrically opposite to my own that I feel I must immediately communicate mine to you. In the last twelve months I have used desensitizing paste in more than two hundred cases without the death of a single pulp, to my knowledge. I have exercised care as to where I have used it, never using it unless it was necessary, and never in cases where there was any great doubt as to the advisability of conserving the vitality of the pulp; and while I have had to make two, and occasionally three, applications in some instances, and have frequently had patients complain of a more or less severe toothache immediately following its application, not a single case has so far reported in which the death of the pulp has followed. That others should have results so different from these is quite a surprise to me, and prompts me to thus communicate my experience to you."

Dr. J. V. Conzett says: "In regard to desensitizing paste, let me say that I have had none of the trouble you speak of in your letter. I had one pulp that ached after the application, and I devitalized it; but that is the only one, and the patient was one that I always have trouble with. The probabilities are that in that particular case I would have had the same trouble if I had not used the paste. As you know, I am using the stronger paste than the one you have on the market; and you will remember that

I advised you to put out the stronger one, but you thought best not to do so. You may say that I unqualifiedly indorse the paste, and use it constantly."

Dr. Chas. E. Bentley writes as follows: "I have recently heard some mutterings concerning the effect of your desensitizing paste upon the pulps of teeth. Having been favored by you in being among the first to use it, my experience may be of some interest. I have to date used it in 130 cases; in one case, a bucco-occlusal cavity in a lower second molar, the pulp died after administration. I was particularly careful in opening into the said pulp for the reason that I suspected a minute exposure might have existed before the application of the paste. To my surprise, I had to cut through about one-eighth of an inch of secondary dentin before I reached the pulp, which convinced me that more or less irritation existed before the application of the paste. I am therefore unable to state whether the paste, previous irritation, or both, were the cause of pulpal death. From the experience I have had I am willing to take the chance of 130 to 1 for the benefits I get from the use of desensitizing paste."

I think that it is only fair to the readers of your journal and to myself, who introduced desensitizing paste to the profession, to publish these statements in the next issue of the *Dental Cosmos,* for the benefit of those who are interested. I am interested only in the truth regarding this preparation, and I know that if it be used as first recommended, it will prove a valuable remedy in the hands of careful men.—J. P. Buckley, in *Cosmos.*

*Aseptic Pericementitis.* Because we have no pronounced symptoms of acute pericementitis we are not justified always in concluding that no pathologic conditions are present or may be developing and that so long as our patient enters no complaint that all is well. A blind abscess may be in existence for many years and all the time be infecting the lymph channels and blood streams with slight or no local manifestations. The insidiousness of these conditions ought to make us most careful in diagnosing all suspected areas by every known test. Present careless methods of root canal work and dependence on sterilizing chemicals of more or less easy solubility may possibly bring our root canal therapeutics into extreme disfavor by our medical confreres.

*Asceptic Canal Procedures.* Where is the surgeon today, who after handling such bacteria-laden objects as the levers of an operating chair, or an engine handpiece, etc., would wrap root canal instruments taken from a bracket table, with cotton in his bare hands, and swab out a wound in the abdomen with such an instrument? If a trained bacteriologist were to witness our every-day procedures at the chair, what would be his opinion of our aseptic surgery? As strictly aseptic root canal work is impracticable let us adopt antiseptic methods and materials and do the best we can to avoid introducing septic infection into the apical areas.—C. E. Kells, *Items of Interest.*

*Faneuil D. Weisse, M.D.* Dean of the New York College of Dentistry, died June 22nd, 1915. Dr. Weisse has for many years been a well known and influential member of the National Association of Dental Faculties because of his official connection with this dental school. He was the author of a very important text book on general anatomy, and taught anatomy and surgery in his school. His school had last year the largest attendance of any college in America.

*Apical Infection.* "In my opinion infections can occur about the root of an imperfectly filled canal, but such infection does not always occur. This is no excuse for being careless, neither is it an excuse for using medicated pastes, with the end in view of having the medicine keep the canals sterile indefinitely. I believe it is well to have certain drugs, like thymol for instance, in our root canal filling material, but I do not believe there is any such thing as a 'permanently antiseptic root canal filling.' In regard to the paste from the New Hampshire dentist, will say that anyone who would depend solely on phenol for sterilizing a putrescent canal can not expect us to have much confidence in other remedies which he might suggest; and surely I would not think much of a paste for filling roots, with or without gutta percha, made of alum, thymol and glycerine. Both alum and glycerine are soluble in water."—Dr. Buckley, in *Items.*

*Filling Root Canals with Pastes.* "Possibly root canal pastes have some merit. They may ease the mind of the operator, but herc their service ends. To my mind, there is as much sense in using such a preparation in a pulp canal which contains non-vital pulp tissue, with the idea of bringing about and maintaining asepsis, as there is in the case of the small boy, who persists in eating green apples because he believes in the advertised merits of 'Pain Killer.' Our experience has taught us that they are practically valueless; in fact some of them, in time, make excellent culture media for bacteria."—E. S. Best, in *Items.*

*Tooth Brushing Hour in the Public Schools.* Dr. C. W. Crampton has introduced into the public school exercises of the New York City schools, a tooth brushing exercise following the noon lunch hour. On a specified day it was estimated that 400,000 N. Y. children brought their tooth brushes to school to inaugurate the idea.

*The Amoeba.* Writing in the July *Dental Summary* Dr. Eugene S. Talbot makes the statement that interstitial gingivitis (pyorrhea alveolaris), is not caused by the amoeba.

*Essentials for Local Anesthesia.* Dr. Herman Prinz in a paper read before the Michigan State Dental Society, says that the five essentials of successful hypodermic injection for local anesthesia, are:

1. A solution of active ingredients corresponding with the physical and physiologic laws governing certain cell functions.
2. A carefully selected hypodermic outfit.
3. A complete mastery of the technique.
4. The selection of a proper method for the case in hand.
5. Good judgment of prevailing conditions.

He recommends the following solution of novocaine,

| | |
|---|---|
| Novocain, | grs. x |
| Sodium chloride, | grs. iv |
| Distilled water, | ℥ i |

Mix, and add to each syringe full two drops of adrenalin chloride solution.—*Dental Summary*, for July.

*Filling Small Root Canals.* There are many canals so small and tortuous that even a fine broach will not enter, to any depth at least. In these cases, after the hemorrhage from the larger canals has been checked and the blood removed, the pulp tissue in the small canals can be disorganized by the use of strong solutions of mineral acids or alkalies. The author prefers making a paste of sodium dioxid and absolute alcohol, placing the paste in the pulp chamber over the small canals, and working it down as far as possible with a smooth broach. The alcohol gradually evaporates, when the sodium dioxid can be decomposed into oxygen and caustic soda by placing a pledget of cotton in the cavity moistened with distilled water. After the reaction has taken place, the alkali can be neutralized with a weak solution of sulphuric acid (two per cent.). This process can be repeated until the desired end is attained. There are other means by which the same results can be accomplished, such as the use of a fifty per cent. solution of chemically pure sulphuric acid, strong solutions of potassium or sodium hydroxid, or a mixture of metallic potassium and sodium (Schreier's paste). These same agents can be used to advantage for the purpose of disposing of a remnant of a pulp in larger canals. It is not safe to anesthetize this remnant by means of pressure. The only cases on record to my knowledge, where toxic symptoms have resulted from the removal of a pulp by pressure anesthesia, followed an attempt to anesthetize a remnant of a pulp or in making the second application of the anesthetizing solution.—J. P. Buckley, in *Items of Interest.*

*Cold Air as an Obtundant.* Dr. C. J. Lyons in the *Dental Summary* for July, suggested that a spray of cold air injected onto a bur running in a sensitive cavity will obtund the dentine so that the operation is comparatively painless. The air should be started at the same instant that the bur touches the sensitive dentine, and the two should be kept in constant and consecutive operation during the process of excavating the cavity.

*Peridental Anesthesia.* Sometimes, where abscessed teeth for instance are to be extracted, it is not desirable to inject a solution into the gum tissues for fear of forcing pus into the tissues, the injection may be made directly into the peridental membrane. Take a small Gates-Glidden drill in the engine and drill a canal along the side of the root deep enough to secure a firm seating of the needle, and inject the solution slowly until anesthesia is complete.

*Filling Root Canals.* The filling of root canals is the most exact surgery which the general practitioner of dentistry is called upon to perform, and is perhaps the most important operation of his daily routine. The successful performance of root canal work demands an intimate knowledge of the anatomy of the teeth, and careful and painstaking labor in the extirpation of all pulp tissue. When the canals have been cleansed they should be filled with a substance which is insoluble in the body fluids and which is impervious to moisture.

It is then largely a question of the ability of the individual operator to find canals and to successfully operate upon them. It has been my experience that the careful operator will thoroughly extirpate and fill all root canals that are of considerable size, and will only fail in the extremely small and tortuous canals, and few of these. In such most difficult cases the use of the radiograph is of great assistance. But in case no means are at hand whereby further progress may be made, and such canals are rendered aseptic and filled as far as possible with a permanent root canal filling, the possibility of such canals giving future trouble has always been somewhat a doubt in my mind. When we examine radiographs of a large number of teeth and see how many root canals have been but partially filled by even the most careful operators; and when we note how few of these have undergone a subsequent infection, we can not but feel that nature has means of protection, and that she may be successful in maintaining health when the odds are not too great against her.

In all such cases the use of germicides and dessicating agents should make the canal contents less favorable for bacterial growth and raise the factor of safety. I believe, however, that the final filling of all canals should be made with a material which is insoluble and impervious to moisture.

Had the Egyptians buried the mummified bodies of their ancestors in the banks of the Nile, they would have been no more foolish than the operator who depends upon mummifying agents to take care of considerable amounts of pulp tissue. Such agents should only be used when all surgical measures have failed and the operator is reasonably certain that the remains of pulp tissue are exceedingly small; and even then they are not safe.—R. W. Bunting, in *Items of Interest.*

*Patterson and Brady Toasted.* The Kansas City Dental Society gave a dinner on May 24th in honor of Dr. J. D. Patterson and Dr. W. J. Brady. Dr. Patterson has for many years been the dean of dentistry in this part of the western country and has most creditably upheld the best traditions of his beloved profession, and he rightly deserves all the honors his confreres may bestow upon him. Dr. Brady is a teacher of many years' experience in the Western Dental College, and at present is establishing a private school of post graduate instruction in orthodontia.

*I. B. Willmott dead.* Dr. Willmott, Dean of the Royal College of Dental Surgery of Ontario, Canada, died in the hospital following an operation on his stomach. Dr. Willmott was well known in this country and highly esteemed for his great interest in professional education and for the nobility of his character. He was a man of great character and a kindly disposition that made many friends here who will be greatly shocked to learn of his death. He will be greatly missed in the councils of the Dental Teachers' Association and the National Faculties Association, in both of which organizations he was an active member, and a constant attendant. He was a good man and made a fine record in his professional career as practitioner and teacher.

*British Soldiers' Teeth.* A Canadian nurse at the base hospital in Boulogne, France, writes: "There is one thing that we can't understand about the British soldiers, and that is the frightful state of their teeth. I have never seen so many decayed or false teeth, or so many young men with no front teeth.

Many are sick with indigestion and have left the trenches because of bad teeth. It seems such false economy, to say nothing of the looks of the thing."—*Dominion Journal.*

*Dr. N. S. Jenkins Honored.* Dentists from far and near met at New Haven, May 15th, to welcome back to America Dr. Jenkins. In closing his speech he gave expression to his views of affairs abroad where he has for so long made his home, in the following words: "In Europe, the old order is perishing. Out of this awful welter of passion and misery may come either a barren victory or mutual exhaustion. But whatever may be the outcome, one thing stands sure—some way must be devised to teach the broken nations to live in harmony with each other. May it be given to our dear America, out of her own experience, and through her patience, compassion and self-control, to show to distracted Europe the way to find enduring peace."—*Review.*

*Some Experiences with Alcohol Injections in Trigeminal and other Neuralgias.* W. Harris reports his experience with alcohol injections in neuralgia. He cautions against injection directly into a mixed nerve, such as the sciatic, on account of the motor as well as sensory paralysis it will produce. Many patients who have thus been operated upon have escaped paralysis by their good luck in the operator missing the nerve trunk and injecting around its sheath. He gives a case of his own experience which is instructive as regards this point. He has had experience with some 200 cases of chronic trigeminal neuralgia. The first division is seldom involved alone, the second also usually being implicated. The supra-orbital is the only branch of the first division Harris attacks. Attempts to reach the infra-orbital branches he thinks dangerous and needless. The second division or superior maxillary nerves can be reached with advantage either at the foramen rotundum in the spheno-maxillary fossa, or at the exit of the infra-orbital nerve on the cheek. In the majority of cases the pain is referred to the upper jaw, and the nerve must be attacked at the foramen rotundum; this is difficult, while the other injection is easy. Harris uses two routes for this posterior injection; the first, and preferably, through the cheek in front of the coronoid process and behind the maxilla. If the coronoid process of the mandible comes too far forward or the antrum bulges backward, it may be hard to direct the needle between these parts and in front of the external pterygoid plate so as to enter the spheno-maxillary fossa. In such cases he tries to reach the nerve from behind the coronoid process, passing the needle through the cheek about four centimeters in front of the middle of the internal auditory matus on the line drawn from the incisura notch to the bottom of the ala nasi so as to pass over the bottom of the sigmoid notch on the lower jaw. With this, the needle passes slightly upward and forward and the pterygoid plate is felt for. The injection of the third division at the foramen ovale is much more certain and easy. For this he uses roughly Levy and Baudouin's line.

In over sixty cases in which he has injected the Gasserian ganglion, he has seen, in one, slight diplopia due to sixth-nerve weakness appear immediately, and last for three months. In another case there was temporary vertigo and nystagmus, for which he can not easily account. Almost always, Harris finds loss of taste occur immediately after injections of the third division of the fifth nerve, confined to the anesthetized half of the tongue in proportion to the depth of the anesthesia. This lasts as long as the anesthesia, and he has seen it two years after injection. This occurs immediately, proving that taste fibers from the tongue pass along to the Gasserian ganglion via the third division. He has encountered two patients who suffered from paroxysmal neuralgia in which the paroxysms began in the throat or posterior palatal region on one side, and spread into the ear and in front of the ear on to the cheek and down the side of the neck. In each case no relief was given by an injection of the third division of the fifth, and Harris considers these as cases of geniculate neuralgia such as are described by Pierce, Clark, and Taylor. Post-therapeutic neuralgia should never be treated by alcoholic injection or any other operative measures, as it makes the cases worse, even if they may appear at first to be relieved. He has seen decided benefit in chronic fibrositis and brachial fibrositis from alcohol injection. Migranous neuralgia is rarely benefited, but the post-influenza of periodic supra-orbital neuralgia may be sometimes completely cured by a single injection. It is in chronic trigeminal neuralgia that most cures are obtained.—*Journ. Amer. Med. Association,* per *Amer. Journ. of Surgery,* and *Cosmos.*

*Melting Aluminum.* In melting aluminum previous to casting, new, clean ingots should always be used. The metal should never be overheated, and when fusing, it should be slightly agitated with the end of an ordinary slate-pencil from time to time, and all dross removed, until a smooth, clean surface presents, after which the casting should be made, observing only moderate speed in doing so, as the metal remains liquid for some moments.—H. J. Goslee, *Dental Review.*

*The Retention and Burnishing of Matrices for Porcelain Inlays.* In the cavity preparation, I aim to get at least two walls parallel to each other, and when possible, I shape the cavity so that the inlay, when finished, will slide in from only the direction from which the stress of mastication is exerted.

In burnishing the matrix, the platinum is carried to place between two pieces of thin china silk, which enables one to carry the platinum to the bottom of the cavity without tearing it. In burnishing, always begin by using a rotary motion near the margins of the cavity, gradually working down into the deeper portions, or, in other words, sort of spinning the platinum into place. The matrix will sometimes break in the bottom of the cavity or in some important retention angle. These breaks can be patched by placing a small piece of platinum over that portion and burnishing it into place; solder with a small thin piece of pure gold; I use a little piece unrolled from a Rowan's or Pack's cylinder. After soldering, the matrix is carried to place, reburnished and finally swaged to place with sticky wax. The wax is put into the matrix hot enough to stick to the platinum, then allowed to cool to a thick putty consistency, when it is pressed firmly to place. This swages the matrix and eliminates the danger of distortion in its removal. The wax is burned out by laying the matrix on a block of clean charcoal, wax side up, and bringing it to a white heat with a blow pipe. Shellack varnish is used on all margins of the matrix, which, as soon as the varnish is dried, is filled with porcelain and baked.—F. H. Skinner, in *Review*.

